# Extravasational side effects of cytotoxic drugs: A preventable catastrophe

**DOI:** 10.4103/0970-0358.44923

**Published:** 2008

**Authors:** Jagdeep S. Thakur, C. G. S. Chauhan, Vijay K. Diwana, Dayal C. Chauhan, Anamika Thakur

**Affiliations:** Department of Plastic and Reconstructive Surgery, I. G. Medical College, Shimla, Himachal Pradesh-171 001, India; 1 Department of Pharmacology, I. G. Medical College, Shimla, Himachal Pradesh-171 001, India

**Keywords:** Cytotoxic, drugs, extravasation, prevention, side effects

## Abstract

In addition to their therapeutic effects on malignant cells, cytotoxic agents have the potential of causing destruction of healthy, normal cells. Extravasation of the drug can produce extensive necrosis of the skin and subcutaneous tissue. Management of these extravasational effects differs from one centre to another and prevention is usually strongly emphasized. We analyzed our management of 12 patients referred to us over five years with extravasation of cytotoxic drugs and reviewed the literature for different approaches with regard to prophylaxis and management of extravasational effects.

**Materials and Methods:**

This study was done in the department of plastic surgery of a medical college. Five years of retrospective data were studied of patients referred to our department with extravasation of cytotoxic drugs.

**Results:**

We managed 12 cases referred to our department with extravasation of cytotoxic drugs. Mitomycin C was used in seven cases (58.33%), vincristine in two cases (16.66%), 5-Florouracil in another two cases while doxorubicin was responsible for extravasational side effects in one case (8.33%). The size of necrosis ranged from 3.75 cm^2^ to 25 cm^2^ with average size of 9.6 cm^2^. In terms of the area involved, the dorsum of the hand was involved in five cases (41.66%), the wrist in another five cases (41.66%), and the cubital fossa in the remaining two cases (16.66%). All cases were treated with daily debridement of necrotic tissue, saline dressing, and split skin grafting.

**Conclusion:**

Extravasation of cytotoxic drugs further increases the suffering of cancer patients. This catastrophe can only be avoided by vigilance and immediate application of antidotes. Once the local toxicity of the drugs takes effect, morbidity is unavoidable

## INTRODUCTION

The role of cytotoxic drugs in the treatment of malignancy is well established and increasing day by day. In addition to their therapeutics effects on the malignant cells, cytotoxic agents have the potential of causing destruction of healthy cells. Due to the relatively low number of cancer treatment centres, it is often not possible for a patient to take the complete course at the centre itself because of financial constraints and long distances from their home towns or villages. After the initial one or two courses, many patients find it convenient to take the remaining treatment at their nearest health institution. However, physicians in such nonspecialized centres may not be aware of the local side effects of the drug. Very often, even in oncology hospitals, the work of infusion of cytotoxic drugs is left to a junior house surgeon or an intern, whose inexperience in venupuncture and ignorance of precautions for infusing a cytotoxic drug can lead to extravasation of the drug.

Extravasation of the drug can produce extensive necrosis of the skin and subcutaneous tissue. This not only adds to the misery of the already seriously ill patient, but can also cause serious functional loss, as most often, the forearm and hand veins are used for infusion. We analyzed our management of 12 patients referred to us over five years with extravasation of cytotoxic drugs and reviewed the literature for different approaches with regard to prophylaxis and management of extravasational effects.

## MATERIALS AND METHODS

**Figure 1:**
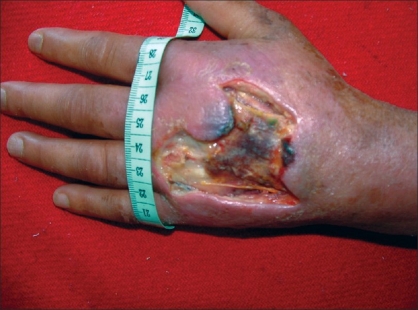
Extensive tissue necrosis of the dorsum of hand

This study was done in the Department of Plastic Surgery of a Medical College in the period from January 2002 to December 2006. Five years of retrospective data was studied of patients referred to our department with extravasation of cytotoxic drug. This is the only hospital in the state providing plastic and reconstructive surgery. There were twelve patients [Table 1] out of which eight (66.66%) were female and four (33.33%) were males. They were aged 48–69 years, with a mean age of 59.8 years. All the patients were from rural areas and belonged to lower socioeconomic strata. None of the patients was educated beyond middle school. Mitomycin C was used in seven cases (58.33%), vincristine in two cases (16.66%), 5-Florouracil in another two cases while doxorubicin was responsible for extravasational side effects in one case (8.33%). The size of necrosis ranged from 3.75 cm^2^ to 25 cm^2^ with average area of 9.6 cm^2^ [Figure 1]. In terms of the area involved, the dorsum of the hand was involved in five cases (41.66%), the wrist in another five cases (41.66%), and the cubital fossa in the remaining two cases (16.66%). There were two cases with erythema and the remaining ten cases had dry tissue necrosis. None of the patients had any nerve, vessel, or bone exposure. In all the cases, chemotherapy was given at the regional cancer centre situated in our medical college and immediate management consisted of hot or cold compresses only. All the patients were referred to us 10–14 days after extravasation with the onset of skin erythema or necrosis. In our department, two cases of erythema were treated by daily saline dressing but they had symptoms of necrosis within a week. In the ward, all the cases with signs of necrosis were instantaneously managed by repetitive debridement of necrotic tissue (skin and subcutaneous tissue) and daily saline dressing with an antibiotic ointment, if infected (two cases).

**Table 1:** Cases with site and size of the lesion

*S. no.*	*Age (Years)*	*Sex*	*Site*	*Size (cm^2^)*
1.	62	Female	Dorsum of hand	2 × 2.5
2	48	Female	Dorsum of hand Laterally	1.5 × 5
3	60	Female	Wrist, Forearm	3 × 2.5
4	63	Female	Cubital fossa	5 × 2.5
5	56	Male	Dorsum of hand	3 × 2.5
6	55	Female	Dorsum of hand	2.5 × 2.5
7	63	Male	Cubital fossa	5 × 5
8	62	Female	Wrist	1.5 × 2.5
9	55	Female	Wrist	2 × 3
10	58	Female	Dorsum of hand	4 × 5
11	69	Male	Wrist	2.5 × 2.5
12	67	Male	Wrist	2 × 4

## RESULTS

In this retrospective analysis of patients who had presented to us in the last five years (Jan 2002 to Dec 2006) with extravasational side effects of cytotoxic drugs, there were eight female and four male patients

**Figure 2:**
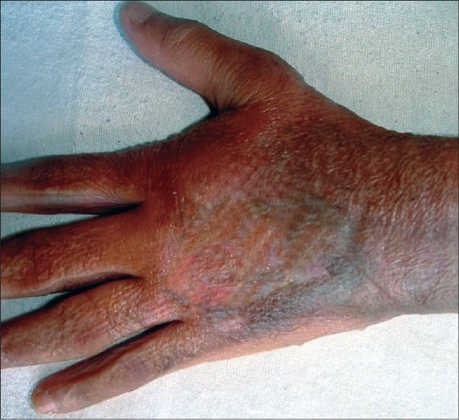
Well healed ulcer after split skin grafting

Within 4–6 weeks, seven cases showed healed ulcers while two cases needed split thickness skin grafts [Figure 2]. Three patients did not come for follow-up. As there was no tendon, vessel, nerve, or bone exposure in any case, there was negligible loss of functional movements of the fingers, hands, or limbs. This slight loss of movements was managed by physiotherapy and pressure garments.

## DISCUSSION

The incidence[1,2] of extravasations in adults is 0.1–6.5%. Drugs which cause tissue damage in general are divided in three groups:[3] (i) Hyperosmolar agents, e.g., concentrated potassium and calcium solutions, hypertonic solutions for parenteral nutrition, and certain radiographic contrast media; (ii) Ischemia-causing agents, e.g., vasopressors, adrenaline, noradrenaline, dopamine, and dobutamine; (iii) Those that are directly toxic to the tissues, e.g., anticancer drugs, sodium bicarbonate, and sodium thiopentone.

The etiopathogenesis of tissue damage by extravasation of drugs enumerated in literature[2,4–6] includes: (1) direct cellular toxicity of drug, (2) vasoconstriction leading to ischemic necrosis, (3) osmotic damage, (4) extrinsic mechanical compression by large volumes of extravasated solutions which again leads to ischemic necrosis, (5) superimposed infection if skin sloughing occurs.

Cytotoxic agents have been classified as irritants or vesicants [Table 2].[1,7,8]

**Table 2:** Vesicants and irritants anticancer drugs

**Vesicant drugs**
Alkylating agents: Mechlorethamine (Mustine HCl)
Antitumour antibiotics (Anthracyclines): Mitomycin C,
Daunorubicin (Rubidomycin), Doxorubicin (Adriamycin),
Epirubicin, Idarubicin, Actinomycin D (Dactinomycin)
Vinca alkaloids: Vincristine (Oncovin), Vinblastine, Vinorelbine
Taxanes: Docetaxel, Paclitaxel
**Irritant (Nonvesicant) drugs**
Alkylating agents: Cyclophosphamide, Ifosfamide, Melphalan,
Carmustine, Dacarbazine, Thiothepa
Antimetabolites: Methotrexate, 5-Fluorouracil (5-FU), Cytarabine
(Cytocine arabinoside), Fludarabine, Gemcitabine
Antitumor antibiotic: Bleomycin
Epipodophyllotoxin: Etoposide
Platinum analogs: Cisplatin, Carboplatin, Oxaliplatin

Vesicant drugs: These drugs cause blister formation which can lead to tissue death and finally ulcer formation.

Irritant (Non Vesicant) drugs: These drugs cause irritant effects on the injection site; pain is the main symptom. If a large amount of the drug is extravasated, ulceration can also be seen as one of the sequalae.

Extravasation of cytotoxic drugs leads to symptoms which are self-explanatory for this catastrophe. The majority of the patients will complain of excruciating pain and itching in the infusion site. Within a few hours, the extravasation area will show erythema, edema, and induration. Within a few days, these signs and symptoms will increase and the skin will show discoloration and desquamation of the epidermis or blister formation will follow. If a large dose of a cytotoxic drug is extravasated or no intervention is taken at this step, the area will show ischemic changes and ulcer formation will be inevitable.

### Management of extravasation

It is well said, “Prevention is better than cure” and this holds true for extravasation injuries also. Once there is an extravasational injury to the tissue, morbidity is inevitable regardless of the management approach taken.

a) Immediate bedside management: Once there is any suspicion of extravasation of a cytotoxic drug, the following steps should be followed:

- Stop the infusion of drug at once.
- Start normal saline infusion in the same IV line (if the canula is patent) as the normal saline will dilute the extravasated drug.
- Send an urgent call to the extravasational injury specialist or plastic surgeon.
- Elevate the limb.
- A parenteral strong analgesic (selective COX-2 inhibitors, ketorolac, diclofenac sodium, nimusulide) will help in the reduction of the inflammatory reaction and its symptoms.
- Apply a cold or hot pack to the limb. Cold packs are used at local site. Hot packs are applied in cases of extravasation of vinca alkaloids as it is presumed that warming of the site will lead to the vasodilatation and diffusion of the drug from the site.
- Intravenous cannulas should be removed in cases of extravasation of irritant drugs whereas they are left in place in cases of vesicant drugs; proper antidotes should be given to the patients.[7]

There are a number of studies and case reports in literature[1,2,9–17] to show the efficacy of various antidotes for these cytotoxic drugs [Table 3]. Oliver *et al.*[9] used topical dimethyl sulfoxide DMSO every six hours for 14 days in 20 patients with extravasation of anthracycline and in the follow-up of 16 patients for three months; no patient developed skin ulceration or necrosis. In a prospective study of 127 patients with extravasation of various vesicant drugs, Bertelli *et al.*[12] used 99% DMSO topically every eight hours for seven days and only one patient had skin ulceration. Dissa *et al.*[13] conducted a study to determine the efficacy of local infiltration with heparin sodium, hyaluronidase, and saline in the prevention of extravasation ulcers due to adriamycin in a rat model and found a decrease in ulcer size. The ulcer size further decreased after additional infiltration of hyaluronidase. Bertelli *et al.*[11] conducted a clinical study on the use of hyaluronidase in seven cases with extravasation of vinca alkaloids and found this to prevent skin necrosis in all treated cases. The use of dexrazoxane in anthracyline extravasation was reported by Langer *et al.*[14] in 2000. They found a statistically significant reduction in skin wounds in mice treated by dexrazoxane. A recent multicentre study has found only one patient who required surgery for skin necrosis out of 54 patients treated with dexrazoxane injections within six hours of extravasation of anthracycline. Scuderi *et al.*[18] reported the use of local injection of saline (20–90 mL) with occlusive topical application of corticosteroids after extravasation to avoid skin necrosis. However, Langer *et al.*[19] found no prevention of skin necrosis by local infiltration of hydrocortisone after anthracycline extravasation in mice models.

In summary, anthracycline extravasation should be managed by DMSO / dexrazoxane / hyaluronidase whereas vinca alkaloids injuries should be managed by hyaluronidase in the doses mentioned in Table 3

**Table 3:** Antidotes and their indications in various cytotoxic drugs' extravasation

Dimethyl Sulfoxide (DMSO)	1–2 mL of 1 mM 50–99% DMSO, TID × 1–2 weeks. Apply topically in the effective area and lead to dry.	Anthracycline and Mitomycin C
Hyaluronidase	150–1500U intravenous or subcutaneous. Contraindicated in infective and cancerous site	Vinca alkaloids, anthracycline, paclitaxel and epipodophylotoxin
Dexrazoxane	1 g/m^2^ within five hours of extravasation, repeated again on second day in same dose and 500 mg/m^2^ on third day.	Anthracycline
Sodium thiosulphate	Mix 4 mL of 10% sodium thiosulphate with 6 mL of sterile water and inject 2 mL of this solution for 1 mg of mechloroethamine or 100 mg of cisplatine in the existing cannula. 1 mL of sodium thiosulphate is injected subcutaneously and repeated several times over 3–4 hours. 0.1 mL of drug should be injected subcutaneously around the leakage site.	Mechloroethamine (Mustine HCl) and cisplatine

Gault[20] stressed the use of saline washouts and liposuction within 24 hours of the extravasation injury. They reported 86% success in the prevention of skin necrosis in a series of 96 patients with various extravasation injuries. Liposuction is performed by a 4 mm blunt-tipped canula inserted into the subcutaneous tissue in the leakage area. Then, 0.9% normal saline is injected through this canula which causes dilution of the extravasated drug. Liposuction is then performed by removing the subcutaneous tissue and the extravasated drug gets diluted by normal saline. This procedure has been reported to be effective in the prevention of skin ulceration/necrosis.[21] None of our patients received any antidote or underwent liposuction.

b) Early surgical treatment or a “waiting” approach: Even if the above mentioned steps are taken care of, the patient still lands up with skin ulceration which needs interventions by plastic surgery and physiotherapy. The plastic surgical intervention involves two approaches: i) one school of thought advises extensive surgical debridement preferably under a fluorescence microscope within 24 h to one week after extravasation and secondary wound closure.[22–25] von Heimburg and Pallua[26] advised early debridement and flap cover for injuries of the hand and cubital fossa as they had found that debridement within 24 hours and flap resulted in better healing as compared to skin grafting. In a study of 14 patients with extravasation of various drugs, Loth and Eversmann[27] reported better results in patients treated by early surgical intervention as compared to those treated by conservative methods. Khan and Homes[28] treated 17 patients immediately after extravasation and found full recovery without any sequalae.

The second approach involves conservative treatment in terms of daily saline dressing if the wound is not infected. The wound is then covered by split skin grafting or a flap if the tendon, vessels, or nerves are exposed. In some cases, amputation is needed if necrosis has involved peripheral vessels and nerves. Surgical intervention[18,29–33] is advised in this approach if erythema, pain, or swelling are not resolved or if there is a large defect due to a skin ulcer or necrosis. In our study, we followed a conservative approach and out of 12 cases, only nine patients continued the follow-up, hence, constituting a small sample size. Although the incidence of malignancies is higher in males, the incidence of extravasation was higher in females (eight females out of twelve patients) in our series. This could be because intravenous cannulation is difficult in females as veins are not prominent

Our institution is the only tertiary care centre for oncology and plastic surgery in the state. The state has far flung hilly areas with poor connectivity by roads. The people are poor and live in the villages. These compounding factors were responsible for the poor follow-up.

There are reports of the use of granulocyte macrophage colony-stimulating factor (GM-CSF) in the management of ulcers in anthracycline extravasation which has shown good results in experimental animals and humans.[34–36]

c) Management of late complication: The late complications of extravasation include disability of limbs in terms of functional and neural (sensory and motor nerves) loss and can be managed by hand surgeons and physiotherapists. As split thickness skin grafts have a tendency for contracture, patients managed by this approach have greater disability as compared to those with flap cover. Secondarily, the split skin graft can adhere to tendons leading to further deterioration in functions. The local flap cover will also be helpful in providing sensation to the raw area which is not feasible with split skin grafts. None of our patients had late complications in the follow-up period.

### Proposed guidelines for the prevention of extravasation

A number of guidelines have been mentioned by many authors in literature.[3,7,10,37] We propose the following guidelines, based on our experience as well as a summary of literature:

- The infusion should be started by experienced staff with the knowledge of extravasational side effects and their management.
- All the emergency drugs and antidotes should be kept at the patient's bedside.
- The nurse and the patient should be fully informed about the symptoms of extravasation and its complications.
- The infusion should be started in the forearm as the muscles will protect the nerves and vessels from injury in case of extravasation. The use of veins on the dorsum of the hand, wrist, and cubital fossa can lead to injury to the nerves, vessels, ligaments, and bones during extravasation as these structures lie without any protection from muscles in these areas. Moreover, as these areas are involved in the movements of the limb it is relatively easy for the IV canula to get dislodged, leading to extravasation.
- Place an intravenous canula in the vessel in a single attempt; secure it with transparent adhesive tape, never use a butterfly cannula.
- First run normal saline or 5% dextrose in the intravenous line. This will not only hydrate the patient—a prerequisite for chemotherapy, but will also be confirmatory for the proper placement of the cannula in the vein as the fluid will not run at full speed in case of improper placement.
- Do not start infusion in the limb with a history of either intravenous catheter insertion distally within 48 hours, or numbness, and radiation or lymph node clearance in the proposed limb.
- If possible, use central venous catheter for infusion.
- Avoid the use of high-pressure infusions.
- Patients should alert the staff on burning pain, itching, stoppage of infusion, and edema in the limb.

## CONCLUSION

Extravasations of cytotoxic drugs further increase the suffering of cancer patients. This catastrophe can only be avoided by vigilance and immediate application of antidotes as once the local toxicity of the drugs takes effect, morbidity is unavoidable
